# Community treatment orders and the experiences of ethnic minority individuals diagnosed with serious mental illness in the Canadian mental health system

**DOI:** 10.1186/s12939-014-0069-3

**Published:** 2014-09-06

**Authors:** Magnus Mfoafo-M’Carthy

**Affiliations:** Lyle S. Hallman Faculty of Social Work, Wilfrid Laurier University, 120 Duke Street West, Kitchener, ON N2H 3W8 Canada

**Keywords:** Community Treatment Orders (CTOs), Mental health, Mental illness, Ethnic minority, Racism, Mental health treatment

## Abstract

**Introduction:**

The prevalence of Community Treatment Orders (CTOs) in the Western world has generated considerable discussion regarding best practices in the outpatient treatment of the seriously mentally ill. Although problems encountered by ethnic minority communities in the various health care systems have been studied to some degree, there is an acute dearth of information on the effects of CTOs on minority individuals. This paper presents findings from research on the lived experiences of individuals from ethnic minority backgrounds who have been the subjects of CTOs in Toronto, Canada, and their perceptions of its impact on their lives.

**Methods:**

Using a qualitative phenomenological approach, in-depth semi-structured interviews were conducted with individuals who have experienced CTOs. Purposive sampling was used to recruit participants (n = 24) from ethnic minority background in Toronto, Canada.

**Results:**

Participants perceived both positive and negative impacts of CTOs. The positives included affirmation of experiences with the mental health system; improved rapport with the case management and clinical team, increased medication compliance and feelings of empowerment. The negative feedback included feelings of being coerced and the stigma associated with it.

**Conclusions:**

The findings of this study suggest that although CTOs are not a panacea for every mental health problem, they can be effective with a specific group who choose to follow through with the expectations of the treatment. The author, however argues that for these individuals to be on a CTO before getting better treatment, brings to the fore a number of issues with the mental health system. This is particularly concerning as it pertains to individuals of ethnic minority background.

## Introduction

A large body of research reveals that individuals of ethnic minority background experience inadequate mental health care, insufficient access to care, and racism in the health care system [1-6]. Studies have shown that support and treatment for this population is not only inadequate in terms of access, it is also frequently insensitive or blind to individuals’ socio-cultural beliefs and preferences, and this may be attributed to factors that include racism and oppression [7-9]. According to a review of the 2007 Mental Health Act in the UK, Blacks and visible minorities were disproportionately more likely to be placed on Community Treatment Orders (CTOs) and treated against their will than those of the dominant racial group [10]. Ethnic minority individuals in the mental health system experience institutionalized racism, interpersonal racism, and direct exposure to race-based abuse and exclusion [7,11].

The prevalence of racism in the health care system is not only evident in the UK, but around the Western world [12]. According to Tinsley-Jones [8], racism tends to create mistrust, fear, and wariness of Blacks, which is further perpetuated by prejudice, misunderstanding, and misconceptions. Racism has implications for health care as it contributes to disparities in the form of inequitable provision of resources in the community [13-16].

Research has shown that the physical and mental health of individuals from minority communities is impacted by a health care system that is shaped around the needs of the dominant culture [17,18]. Studies demonstrate that mental disorders are on average more widespread among Blacks than Whites, and that options for treatment are often influenced by race and socio-economic disparity [19]. For example, mentally ill individuals from ethno-racial minority backgrounds are more likely to be prescribed antipsychotic medication and less likely to be offered psychotherapy [20,21]. Furthermore, individuals from ethnic minority backgrounds have higher dropout rates or non-compliance with treatment compared to the dominant group. This is attributed to adverse experiences in the mental health system and the cultural incompetence and insensitivity of service providers [7,22]. Based on the fact that minority individuals have less access to services than the dominant group, the tendency is high for them to seek mental health care later and they are often misdiagnosed, which results in the likelihood of receiving inadequate treatment [23,24].

While the experiences of ethnic minority individuals in the health care system have been documented, albeit in a limited way, their experiences with CTOs remain invisible. CTOs were introduced to provide treatment for seriously mentally ill individuals deemed “hard to treat”. These individuals are typically those who are less likely to maintain the gains achieved in hospital. In Ontario, the enactment of Bill 68 in December 2000 led to amendments to Ontario’s Mental Health Act and the introduction of CTOs as a prescribed treatment option for the persistently mentally ill. It is viewed by many as a means of providing care and treatment for individuals diagnosed with serious mental illness that is less restrictive and intrusive than involuntary hospitalization [25]. CTOs authorize legally mandated outpatient treatment for individuals diagnosed with severe or serious mental illness usually requires that the subject of the order interact with a variety of service providers including physicians, case managers, and in some cases, stakeholders. Torrey and Zdanowicz [26] describe CTOs as a treatment option that legally mandates a person to follow through with an established treatment plan or “risk sanctions for non-compliance such as potential involuntary hospitalization and treatment” (p. 337).

The Ontario Mental Health Act [27] states that the purpose of a CTO is “to provide a person who suffers from a serious mental disorder with a comprehensive plan of community-based treatment or care and supervision that is less restrictive than being detained in a psychiatric facility” (MHA 33.1(3)). A CTO serves as a legally binding agreement between a person diagnosed with serious mental illness and his/her physician; the order outlines the specific treatment, care, and supervision to be provided in the community. In Ontario, amendments to the Ontario Mental Health Act in 2000 included the introduction of CTOs as a prescribed treatment option for the persistently mentally ill.

CTOs have received controversial reviews with as many opponents as supporters. Supporters argue that CTOs stem the tide of the ‘revolving door syndrome’, where individuals diagnosed with serious mental illness are provided with adequate treatment in the community instead of being repeatedly admitted to an inpatient unit. They believe such mandated treatment prevents these individuals from falling through the cracks of the mental health system. They argue that CTOs are a humane way of providing support, treatment, and resources for individuals who otherwise would not receive the benefits that can be derived from treatment [28,29]. In addition, the process has been shown to reduce encounters with the criminal justice system for some of these individuals [30]. CTOs are also perceived as an effective way to offer much needed help to individuals who are judged a danger to themselves or others if they do not receive intervention [31,32].

Critics, on the other hand, argue that CTOs should not be seen as a panacea for effective outpatient treatment, emphasizing that alternative options for people with mental illness are more appropriate and in the best interests of these individuals. Some have described CTOs as coercive and unethical because they allow health care practitioners to seriously infringe upon the human rights of individuals [33,34]. Others point out that they are a form of forced treatment that eliminates individual autonomy in decision making [35,36]. Others contend that CTOs discourage mentally ill persons from seeking help from professionals for fear of being mandated to receive treatment.

The literature on CTOs also shows most of the studies focus on the dominant group. While studies that address issues pertaining to ethnic minorities and their experiences with the CTOs are hard to come by, a study with the Maoris [37] seems to be an exception. In this study, the researchers explored the impact of CTOs on Maori patients, their extended family and view of health professionals in New Zealand. The physicians in the study expressed concern regarding the conflict reconciling traditional beliefs with the medical model. Also, an evaluation of CTOs in Ontario in 2005 revealed the lack of cultural perspective regarding the treatment of individuals diagnosed with serious mental illness. The reviewers strongly recommended that clinicians and researchers explore and include attention to culture in the treatment of the mentally ill. Despite these recommendations, little change to the status quo has been observed [38].

The research from which this paper draws was designed to engage this dearth in knowledge and address the gaps in the literature. The author, who is a person of ethnic minority background himself, has been a social worker in the mental health field for more than ten years. During this period he worked with hundreds of individuals living with serious mental illness in the context of the large and culturally diverse Canadian city of Toronto. Many of his clients were the subjects of CTOs. The lack of knowledge and research in the above review heightened the persistent concerns about the experiences of ethnic minority individuals in the mental health system, especially those who lived under the mandate of CTOs. This prompted the desire to embark on the research to explore CTOs from the perspective of individuals of ethnic minority background.

The research questions the study explored were 1) What effect do CTOs have on ethnic minority individuals and how do these individuals perceive the treatment? and 2) How are CTOs experienced in comparison with other experiences in the mental health system? Phenomenology was used as a conceptual framework best suited to effectively explore these questions because the objective of the study is to explore the lived experience of the participants from their perspective and understand the meanings they constructed from it. As Creswell [39] asserts, a phenomenological study “describes the meaning of the lived experiences for several individuals about a concept or phenomenon” (p.76). More than a conceptual framework, however, phenomenology is also a methodological approach that probes the human experience, shedding light on the complexity of individual perceptions and offering ways of gaining insight and understanding into people’s experiences. This will be described in subsequent sections.

## Methods

### Setting

The study was undertaken in Toronto, Canada. It aimed to explore in detail the encounters of individuals of ethnic minority background who had experienced CTOs. Twenty-four individuals of ethnic minority background were recruited through information flyers that were given to clinical coordinators and case managers for distribution to their clients in the community. Flyers were also left at the offices of physicians and psychiatrists. Prospective participants for the study were also recruited from most of the hospitals in the greater Toronto area. These included the Centre for Addiction and Mental Health (CAMH), The Scarborough Hospitals (the General and Grace campuses), Rouge Valley Centenary Hospital and the North York General Hospital (NYGH). Approvals for the study were granted by the Research Ethics Boards of both the Centre for Addiction and Mental Health (CAMH) and the University of Toronto, Ontario.

#### Participant selection

The criteria for eligibility required that a participant should: 1) be over the age of 18, 2) have a diagnosis of mental illness, 3) be an individual currently on a CTO or the subject of a CTO in the past, and 4) be of ethnic minority background. Study participants were assured of confidentiality and anonymity and were given an honorarium of $20.00 for their participation. Interested participants who met the eligibility criteria were asked to contact the researcher by telephone, at which point the study was explained in more detail and a mutually convenient appointment was arranged for an interview. Participants with no telephone access were advised to contact the researcher through an intermediary, which was either their case manager or community worker. Participants were assured that their participation was voluntary and they could withdraw from the interview at any time without jeopardizing their treatment or access to services. Participants had the option to refuse to answer any question. To protect the anonymity of participants’ identities and the confidentiality of their information, aliases are used instead of real names and identifying information was removed from all research reports and writings, including this paper and the embedded quotes.

#### Participant demographics

Participants in the study were between 18 -59 years, had been diagnosed with mental illness between 1 to 17 years previously and had been on CTOs for periods from a few months to more than three years. Fifteen females and nine males participated. They were members of the following ethnic minorities: Blacks (Black Canadians, African Canadians and Caribbean Canadians) (n = 14), West Asians (n = 1), South Asians (n = 6), East Asians (n = 2) and one person from the Middle East (n = 1). The diagnoses as reported by the participants were schizophrenia (n = 16); schizoaffective disorder (n = 1); depression (n = 2) and bipolar disorder (n = 5).

#### Data collection

Data collection was conducted in ways that are consistent with the phenomenological approach. Interviews were designed as semi-structured in order to probe the human experience, shed light on the complexity of individual perceptions and offer ways of gaining insight and understanding into people’s experiences. The interview process used an informal approach that built rapport and encouraged a good comfort level among participants, in order to facilitate an honest and comprehensive response. The questions explored participants’ day-to-day activities while on a CTO and they were encouraged to discuss their thoughts and feelings about the CTO, about any experiences of racism associated with their mental health treatment and their relationship with the clinical team (physician, nurse, and case manager) while on a CTO. Interviews were audio recorded and transcribed using the services of a professional transcriber.

#### Data analysis

The phenomenological approach uses different ways of analysing the data. For example, it applies both narrative and interpretive approaches, such as the ability to use metaphors of life as narrative, and shift that from personal experiences to situating it socially and culturally. Metaphors were used to interpret participants’ narratives and to link their personal experiences to broader systemic issues. The data were further analysed by using the NVIVO QSR software for coding and identifying themes. The themes and meanings derived from the study’s analysis were utilized to develop the textured and nuanced descriptions of participants’ experiences. The textured and structural descriptions were integrated “into the meanings and essences of the phenomenon” [40].

#### Trustworthiness

The researcher kept a reflexive journal throughout the interviewing process and documented all aspects of the interactions with the participants. This contributed to the trustworthiness of the interpretation of the data. Furthermore, credibility was supported by the prolonged engagement with the data and the regular debriefing with fellow coordinators of the CTO program in Toronto and the author’s supervisor.

## Results

The overarching finding of this study suggests that participants experienced CTOs both in positive and negative ways. This section will present the overview of the results under the two major themes categorized as positive and negative experiences. Several subthemes are clustered under each major theme.

### Positive experiences

#### Support

The initial reaction to CTOs by some participants was rooted in the fear that the treatment would hold them back from what they intended to achieve in life. Subsequent to receiving treatment under the CTO and experiencing support, however, their perception tended to be more favourable.

Initially that’s how it feels like, it feels scary. It feels like somebody is going to hold you back and stop you from doing what you want to do and you’re told what to do but as you go through it, they let you realize that you have absolutely all the freedom to let go and get out of it. And then you start to realize that they do help and support you so you kind of look forward to having CTO. (Dana)

Most participants expressed appreciation for the CTO they had experienced, stating that it provided a form of protection: a support system that enabled them to stay grounded and responsive to treatment. Some participants acknowledged the importance of being supported by a case worker who checked in with them periodically. Most of them looked forward to the visits of their case worker. Some described it as a catalyst to improving their health. According to the participants, prior to the CTOs they seldom saw or met with their psychiatrist, because they did not show up for appointments and no one followed up with them. The existence of the CTO for some participants meant having the support of someone they could trust, depend on, and look up to. The following quote in reference to a CTO case manager elaborates on this theme of support:

I think it was a good idea mainly in that I realized that being on a CTO gave me some sort of support because it made me realize that they were really trying to help me. I had a nurse (name of worker) whom I liked very much and who was very helpful to me. She was very concerned about my condition and she would come once a week and I really kind of looked forward to her visits. And then I was seeing the psychiatrist more often… (Margaret)

Others talked about the issue of medication non-compliance. They believed CTOs ensured that they received support and direction from their workers in the community.

Well, definitely being on a CTO did help me a lot because I had a tendency to not take my medication properly and I was scared of medication. I totally needed a lot of support to take my medication and I was very scared of the doctors and it was sometimes scary for me to attend appointments with the doctors. I used to get very anxious and confused and nervous. It felt like some examination was coming over for me and I had to prepare for it. But being on a CTO, the doctors made sure that I regularly attended my appointments as well as took my medication, day and night and not only was it about medication and appointments, I also felt that it was about taking care of my diet and my personal activities as well. (Dana)

#### Organization

Some of the participants discussed being “well in my (their) routine” and described their current ability to organize themselves and take their medication regularly; they found themselves in a better position to meet with their mental health workers, whose involvement kept them grounded and organized. They also talked about assignments and other activities that required going to the library and attending support groups. This is something they had never done when not under the CTO. Making the effort to integrate the demands of the treatment into their daily activity speaks to the effectiveness of the program in the lives of the participants.

When I get up in the morning it feels like I’m well in my routine with my medications, my appointments, my social worker visits, I’m active in my brain. I don’t slug down, I don’t want to sleep the whole day. There’s definitely a lot to do; (name of worker) leaves me with a lot of homework to go to public libraries and to make charts and there are assignments of my health care and to get to know myself better and I have instructions from the doctors and the hospital to get to know my diabetes very well and attend an education program to know my diabetes at the hospital. And then I’m told to exercise and go to (Name of program) and I’m also told to take my medication properly and to work on my diet. So I’m really busy with CTO and everything because sometimes you can get lazy so CTO makes sure I’m not lazy. (Evelyn)

Continuing with the theme of organization, some participants reflected on how being organized and going through their daily routines in a well-structured way enabled them to find and hold down jobs and lead a normal life. In addition to medication compliance and the improved ability to get along with others, some of the participants discussed the importance of the treatment associated with the CTO: the CTO stabilized their condition, and the relationship with the case management team enabled them to live productive lives.

Yeah, it’s very, very, very good. Without them I wouldn’t be able to do the things that I do. I can’t get a job. They help me get a job. I have worked at (name of employer) for five years now. Then we slowed down, so I came home. They called me back to work, but I have a knee problem, so when I recover, I will go back to work. That’s the information I have for you for now. (James)

With their enhanced ability to organize their mundane activities and lead productive lives, participants reported that their feelings about self and self-worth were improved. They were able to reflect on their process as they described their experiences prior to CTO, during the CTO process, and their progress, which empowered them and enhanced their self-esteem.

#### Rapport

Another major theme in participants’ positive experience is the rapport that developed between them and the clinical team (psychiatrists, nurses, and the case management team). Participants acknowledged and described the significant role of the clinical team with regard to the CTO. Most participants had case management support and described their interaction with their case managers in positive terms. According to some of the participants, interaction with their case managers was very positive; they had good rapport and they felt comfortable and were able to communicate freely with their worker. Essentially, the worker helped them to feel good about themselves.

Oh well, my case manager is very good. He’s very supportive, very understanding. He’s a good communicator. I don’t feel…he doesn’t make me feel like a patient. He makes me feel good so by him doing that it reflects on the CTO that it’s a good thing that they’re doing in the community… (Alice)

Another participant expressed satisfaction regarding interactions with his worker, describing these as a “positive experience”. The supportive relationship has been beneficial, and as a result, his condition improved.

Well it was very positive and very cooperative and they also feel, like my caseworker is (name of worker) and she feels very happy interacting with me. (Ben)

#### Racism

The study attempted to explore the lived experience of participants in terms of their social location as people of colour to determine whether or not they felt CTOs discriminated against or exhibited any racial undertones. Most participants neither thought that the treatment had any racist implications, nor did they perceive the decision to assign the CTOs to be race-based. They believed that being from an ethnic minority background had nothing to do with the treatment they received. These participants asserted that CTO was the best treatment for them at that particular juncture of their lives.

Also, according to most participants, experiences during hospitalizations did not reveal any racial undertones. For example, some participants pointed out that treatment was provided by nurses and doctors of all races and they did not feel that their treatment was any different from those of White patients.

Well, I don’t really think about ethnicity being on a CTO. It’s just when I was placed on a CTO, this was not taken into account. I was just placed on it because I was not taking my medication; I was a patient, so my ethnicity was not taken into account. (Alice)

I don’t feel, I never really do feel since I got sick. I didn’t really feel like it’s affected me in a negative way. If anything, it’s been positive. I mean, when I go to the hospital, there are black nurses and white nurses. I don’t see them treat me or anyone differently. I have not experienced any racism or anything like that from anyone that I’ve come in contact with since I’ve been sick, you know. (Frances)

I thought it might have had something to do with me going to the hospital to begin with, but once I was there, I didn’t feel that way, because I was brought to the hospital by the police and I know the police and black people in this country haven’t always gotten along (laughter); and me in general dealing with the first few officers that came, you know, it’s not something that I exactly wanted to do or, but I had to and that’s, you know, yeah. (George)

### Negative experiences of CTOs

#### Racism

While the majority of participants experienced race as being fair with regard to their CTOs, there were a few who experienced the negative effects of racism. This group believed that their CTO treatment was racist and the reasons they gave ranged from personal to broader structural issues. Some participants pointed out how the majority of those on CTOs are racial minorities, thus implicating structural factors. Indicating micro processes and personal factors, others felt they did not benefit from the treatment provided under the CTO and that the treatment and support was inadequate. More specifically, they reported that they were not offered talk therapy or counselling while on the CTO, which they would have preferred to seeing a psychiatrist and/or taking medication. The following quotes exemplify both structural and personal reasons:

It is… CTO is a racist thing. Do you want to know why CTO is a racist thing? Because of the majority of minorities that are on CTOs, they are either African-Americans or Hispanic and or they are from some third world country… (Lawrence)

I think so (CTO is racist) because I wasn’t offered any other therapy like cognitive therapy or anything else like that because I’m not sure whether it was because of my race or because they were limited on finances. But I would have preferred talk therapy or something more in depth than just going in to see the psychiatrist and not really doing anything except telling him you know, giving him an update of what was happening in my daily life, you know, without any kind of response. So, I would have liked more interaction with the therapist… (Oscar)

#### Stigma

Analysis of the interviews showed that some participants had negative perceptions of CTOs because they felt it stigmatized them and made them feel like second-class citizens. This view was also held by participants who expressed optimism about the treatment; they reported they were treated differently while in the hospital and in the community, which they attributed to the CTO. For example, one participant described a situation in the hospital where the psychiatrist reassured a nurse that the participant was not violent and talked about her as if she was not present. In this case, it was assumed that the client did not understand what was going on around her.

…maybe once at the hospital, a nurse, was talking about me. I was in the room and the doctor was telling her to bring me to the tenth floor and she was talking negatively about me like I was going to hurt her or, you know, I’m violent. And I heard the doctor say ‘She’s not going to hurt anyone. She’s just hearing voices’. So maybe that was the only experience I had experienced, you know. (Frances)

(Name of worker) brought up a good word the last time, mentalism, yeah, that, like mentalism is like racism, but I haven’t experienced that really, except for that one incident when I went in the hospital to be admitted, the nurse was so scared of me. That’s the only thing. And she was talking about me like I wasn’t even there. But I didn’t talk. I should have said something, but I didn’t. (Frances)

#### Coercion

Participants expressed feelings of being coerced into accepting the CTO. Most felt that they had consented to the treatment order due to the fear of being kept in the hospital for a longer period. Others expressed that they were uncomfortable with the involvement of the police in their treatment. They talked about how being individuals of colour made it difficult to trust the police. A participant’s description of her experience with the CTO process speaks to the coercive nature of the treatment and how she felt crippled with fear.

Yes that (coercion) happens all the time and like I said before, they (CTO staff) make it seem like they’re going to hold you back and they look like they’re going to control your life and they’re going to take your freedom and they’re going to take you back to the hospital if you don’t take your medication. They’re going to get you arrested. There’s a lot of that in there and it is very scary but if you talk to them about it, you have all the support of the lawyers and all the papers and cards given to you if you want to make a law case against them and you’re free to do whatever you want to do. (Evelyn)

The feeling of coercion is supported by another participant who stated that the threat of her son being apprehended by child welfare services compelled her to cooperate with the treatment recommendations. Though she expressed feeling more at peace as a result of the order, she considered it coercive.

Sometimes I do (sic) (take medication for the sake of) my son, because they told me, if I don’t take the medication, Children’s Aid might take my son, you know, I might lose my son. So I used to think like that, because I said, in the beginning I did, and I didn’t know if I’m just getting a rest from the devil or it’s the medication, because I’ve never had a medication that didn’t let me hear voices. All the medication through the years, you know, it’s just that now, it’s not so evil, it’s not foiling, it’s not telling me to hurt anyone or hurt myself. So, I have a peace of mind for the first time in a long time. (Frances)

While coercion comes in various forms as these narratives attest and as other participants also made reference to the coercive nature of the treatment, for most participants, the major reason for following through with the expectations of the CTO was because it was a way of leaving the hospital. Several participants emphasized that they knew their discharge from hospital was contingent upon consenting to the conditions of the CTO.

## Discussion

Themes generated from the analysis of the data illustrate some differences among the participants with regard to their perspectives on CTOs. In this section, I discuss my interpretations of the participants’ feedback in three parts. The first part explores participants’ experiences with the mental health system- this can be described as the interface between participants and the mental health system. The second part on racism could be described as an intersection with issues of diversity. The third part on coercion and stigma could be considered a form of coercive power.

### The mental health system

The interface between participants and the mental health system is multifaceted as it involves multiple players in provision of treatment for individuals with serious mental illness. Most participants acknowledged the gains made being on CTOs, without which a number of them would probably not have been able to access outpatient mental health support. This would have resulted in these individuals falling through “the cracks” of the mental health system, as proponents of CTOs argue [29].

Medication compliance is an integral part of the CTO process, and this is clearly articulated in the CTO agreement or treatment plan. The outcome of the study revealed the impact of medication on the well-being of the participants. This finding supports prior research on CTOs that found mandated outpatient mental health treatment generally improves medication adherence [41-43].

Also, the study showed that rapport between the ethno-racial minority participants and the clinical team was enhanced during the CTO period. Most participants felt that they were heard and treated with respect and dignity. Practitioners described CTOs as enhancing support for continued contact and stability [29]. In this study, participants clearly articulated the benefits of the treatment and expressed their appreciation of the rapport they developed with their clinicians, which made them feel validated and respected.

However, the data also suggests that the structure of the mental health system may have contributed to the inability of the participants to access effective treatment without a CTO. Case management support as part of the CTO support was identified as playing a significant role in the participants’ lives. Majority of the participants reported a lack of community resources upon discharge from hospital prior to the CTO. However, despite the appreciation of CTOs, it should be noted that most participants did not initially embrace the treatment order. The ambivalence of the participants was reflected by the opposing feelings or emotions toward treatment, and may be attributed to their lack of understanding or familiarity with CTOs. Participants likened treatment to being on probation, even associating it with the criminal justice system. While the impressions of participants have been addressed in previous studies [37,25], no study has specifically explored participants’ feelings of ambivalence.

The phenomenological approach to this study facilitated an examination of the participants feeling about CTOs, which for the most part were reported as positive. The acceptance of the CTO enabled the participants to relate more positively, thus enhancing their self-esteem and increasing their sense of security. This study provides participants with a platform to express their feelings regarding CTOs. The results not only support the findings of other researchers [29], but also shed light on the views of ethnic minority individuals.

### Issues of diversity

The intersection with diversity, arguably brings to light the dearth of literature on CTOs within the context of race and culture. The review of CTOs in Ontario identified concerns around the lack of consideration for diversity and the provision of services for individuals of ethnic minority backgrounds [38]. The study unveils participants’ perceptions in relation to race and its impact on their treatment.

Researchers show that clients of ethno-racial minority backgrounds experience racism in the mental health system, in part due to clients’ socio-economic status and their distrust of the system [24]. Overall, the findings of the current study refute this assertion. Here, most participants believed that CTOs were neither racist nor had any racist implications or ramifications. Research also shows that individuals from minority backgrounds diagnosed with mental illness are less likely to receive adequate treatment, thus making compliance with mental health treatment difficult [23,24]. Yet, these findings are affirmed only in a few participants of the current study who experienced racism with regard to CTOs. This raises serious questions. The fact that the majority of participants argued against racist implication of CTOs demands further critical exploration because it goes contrary to what the literature suggests. One wonders whether participants were exhibiting “political correctness” because they were being interviewed by a CTO clinician or practitioner or were referred for the interview by a physician or community worker who is associated with their treatment.

Feedback from participants presented a measure of the dichotomy often experienced by immigrants [44], thus, owing allegiance to one’s country of origin and the host country as well. Racism, as expressed by the majority of participants, is sometimes counter-intuitive. According to Bhabha [44], the perception is that, a person’s current environment in comparison to the country of origin often presents some difficulty. That is, the feeling of not knowing whether to accept what is being experienced as having a negative impact. Essentially, racism may be contextualized in different forms. The participants acknowledged knowing and having experienced racism. Yet, the surprising finding that the majority of the participants did not associate CTOs with racism may be attributed to a number of factors: first, the fact that participants believed treatment is meted out fairly; and secondly, that they viewed treatment received being better than the resources or the support available prior to the CTOs or better than services offered in their homeland. On the other hand, the issue of racism comes into play when individuals of ethno-racial minority background are made to feel inferior about who they are and the type of resources/services provided to them. In this regard, services deemed appropriate for the dominant group is appreciated by minority individuals whether or not it has their welfare or interest at heart [17,22].

### Coercive power

The legal power of the CTOs, as observed by participants, can be both empowering and disempowering. Participants frequently talked about feeling disempowered by the coercive nature of the process, but somewhat felt empowered by the improved functioning that compliance sometimes facilitated. Existing literature on CTOs does not explicitly discuss issues of disempowerment among participants; rather, most of the studies explored the subject from the perspective of service providers [25]. The insight provided by participants in the current study illuminates the feelings and impressions about the disempowering nature of treatment. In particular, it identifies their perspective as ethno-racial minority participants. Comments like “I need to follow certain guidelines that I was committed to…” speak to the feelings of powerlessness and the need to abide by the strict rules of treatment. Others emphasized the fact that they had to be on their best behaviour, believing that the CTO limited their speech and freedom.

Some participants reported that CTOs did not empower them and thus prevented them from seeking help; they felt controlled by the system in every way. Their lived experience was based on fear that they might inadvertently violate the conditions of their treatment and be apprehended. Some questioned the worth of CTOs in light of this powerlessness. Linhorst [45] postulates that individuals diagnosed with mental illness often experience hopelessness as a result of the feelings of powerlessness, which exposes them to inhumane, emotional, physical, and psychological treatment. For individuals of ethno-racial minority backgrounds, hopelessness in the context of being persons of colour creates multiple levels of oppression [17]. Thus, being of a racial minority entails its own unique issues, which are further compounded when coupled with mental illness and broader issues of power.

The experience of coercion which emerged as a significant theme in this study, supported past studies on CTOs [46,33]. Overall, it is possible that the implementation of CTOs could be ethically questioned; however, the eventual benefit of treatment to participants should not be overlooked.

#### Limitation of study and future research

It is worth noting that this study had a number of limitations. For instance, participants recruited could not be described as a fair representation of the different ethnic minority communities in Toronto. Also, the experiences shared by the participants may be different in other jurisdictions as this was solely based on their experiences in Toronto. In addition, potential participants were eliminated due to their inability to express themselves clearly in English; this was due to lack of financial resources to hire translators.

The researcher is hoping to embark on a larger study that will explore different communities and their experiences with CTOs in a future study. I hope to do a similar study with the dominant group to find out what the similarities are. These proposed projects would depend on the availability of funding.

## Conclusion and relevance

The findings of the study identified a number of themes expressed by the CTO participants of ethno-racial minority backgrounds. Of significance, participants in this study believed CTOs enabled them to access treatment resources which otherwise would not have been available to them. Previous studies have neglected this aspect of CTOs, focusing instead on how the inclusion of case management and medication makes differences in the in the well-being of outpatient mental health clients.

Participants’ perspectives on racism and the cultural context of CTOs also add to the literature through exploring the viewpoint of ethno-racial minority participants. Ambivalence expressed by participants, the role of medication, lack of access to preferred treatments, and stigma reported in this study had previously been addressed in the literature. However, this study presented the perspective of ethno-racial minority participants and their impressions and lived experience of CTOs.

The utilization of a phenomenological study enabled participants to articulate their lived experiences in a way that would not have been expressed by using other qualitative methodologies. Participants discussed the impact of the experience of CTOs on their lived experience as individuals of ethno-racial minority background. The use of phenomenology suited the purpose of exploring the lived experience of participants on CTOs as a peculiar phenomenon. Hence, feedback from participants exhibited the benefits of the treatment despite reports to the contrary. Most participants believed that CTOs enabled and empowered them to follow through with the required treatment. Also, it could be concluded that CTOs somewhat addressed the breaches in the mental health care system where individuals of ethnic minority backgrounds have the tendency of falling through, thereby addressing the needs of individuals deemed ‘hard to treat’ [47].

I believe this study will encourage professionals to not only explore CTOs from the perspective of participants’ rights [33], but also from the effectual perspective of the treatment. Although the argument regarding the coerciveness of treatment is true, as reported by some participants, one could state that the absence of CTOs could result in a substantial number of individuals with mental health diagnosis becoming part of the “revolving door syndrome”; thus, moving round and round to fit the revolving door analogy in the mental health system. I will argue that although it is fair to describe CTOs as coercive and disempowering [33], this also buttresses the argument that CTOs are intended for a subsection of individuals diagnosed with serious mental illness. Overall, the contention is that CTOs are not focused on the racial make-up of individuals but are instead a measure for providing effective treatment to individuals who otherwise would not have benefitted from the mental health system.
